# Exploring the Incorporation of a Novel Cardiotoxicity Mobile Health App Into Care of Patients With Cancer: Qualitative Study of Patient and Provider Perspectives

**DOI:** 10.2196/46481

**Published:** 2023-12-12

**Authors:** Megan E Gregory, Weidan Cao, Saurabh Rahurkar, Pallavi Jonnalagadda, James C Stock, Sanam M Ghazi, Endia Reid, Abigail L Berk, Courtney Hebert, Lang Li, Daniel Addison

**Affiliations:** 1 Department of Health Outcomes and Biomedical Informatics College of Medicine Univeristy of Florida Gainesville, FL United States; 2 Department of Biomedical Informatics The Ohio State University Columbus, OH United States; 3 The Center for the Advancement of Team Science, Analytics, and Systems Thinking (CATALYST) College of Medicine The Ohio State University Columbus, OH United States; 4 Cardio-Oncology Program, Division of Cardiovascular Medicine College of Medicine The Ohio State University Columbus, OH United States; 5 Department of Internal Medicine College of Medicine The Ohio State University Columbus, OH United States; 6 Biomedical Sciences Program College of Medicine The Ohio State University Columbus, OH United States; 7 Division of Cancer Prevention and Control, Department of Internal Medicine College of Medicine The Ohio State University Columbus, OH United States

**Keywords:** cancer, cardiology, implementation science, mobile app, oncology, mobile phone, cancer patient, patient care, mobile health application, application, implementation, design, development, symptom tracking, cardiotoxicity, cancer therapy, symptom, primary care

## Abstract

**Background:**

Cardiotoxicity is a limitation of several cancer therapies and early recognition improves outcomes. Symptom-tracking mobile health (mHealth) apps are feasible and beneficial, but key elements for mHealth symptom-tracking to indicate early signs of cardiotoxicity are unknown.

**Objective:**

We explored considerations for the design of, and implementation into a large academic medical center, an mHealth symptom-tracking tool for early recognition of cardiotoxicity in patients with cancer after cancer therapy initiation.

**Methods:**

We conducted semistructured interviews of >50% of the providers (oncologists, cardio-oncologists, and radiation oncologists) who manage cancer treatment-related cardiotoxicity in the participating institution (n=11), and either interviews or co-design or both with 6 patients. Data were coded and analyzed using thematic analysis.

**Results:**

Providers indicated that there was no existing process to enable early recognition of cardiotoxicity and felt the app could reduce delays in diagnosis and lead to better patient outcomes. Signs and symptoms providers recommended for tracking included chest pain or tightness, shortness of breath, heart racing or palpitations, syncope, lightheadedness, edema, and excessive fatigue. Implementation barriers included determining who would receive symptom reports, ensuring all members of the patient’s care team (eg, oncologist, cardiologist, and primary care) were informed of the symptom reports and could collaborate on care plans, and how to best integrate the app data into the electronic health record. Patients (n=6, 100%) agreed that the app would be useful for enhanced symptom capture and education and indicated willingness to use it.

**Conclusions:**

Providers and patients agree that a patient-facing, cancer treatment-related cardiotoxicity symptom-tracking mHealth app would be beneficial. Additional studies evaluating the role of mHealth as a potential strategy for targeted early cardioprotective therapy initiation are needed.

## Introduction

### Overview

There has been a rapid increase in novel anticancer therapies, with >150 new approvals since 2000 alone [1,2]. Many of these have been associated with dramatic improvements in survival [3,4]. However, concurrently, cardiotoxicity is a potentially severe adverse effect of novel cancer therapies which limits the use of several effective cancer therapies. Cardiovascular disease has become increasingly common among patients with cancer receiving novel cancer therapies, with a reported incidence of up to 38% [5,6]. Patients with cancer who develop concurrent cardiovascular disease, including cardiotoxic arrhythmias, heart failure, hypertension, and myocarditis, have worse long-term quality of life (QOL) and poorer outcomes [1,7]. Yet, most of these events are missed until severe morbidity or death occurs [6,8]. Thus, early recognition of cardiotoxic events in high-risk patients with cancer is paramount [9-16].

Mobile health (mHealth) has been investigated to screen for cancer-related symptomatology (eg, pain and chemotherapy side effects) and to improve QOL and outcomes in patients with cancer. In particular, mHealth apps and symptom-reporting systems are powerful tools to improve QOL, symptom detection, and survival [17-24]. Prior work shows that patients with cancer are willing to use mHealth apps and tend to be compliant with electronic symptom reporting [25]. Yet, to date, there have been no studies examining mHealth for symptom-tracking for cancer-related cardiotoxicity [26]. Given the severe consequences of cardiotoxicity, there may be a role for mHealth in screening for this complication and improving QOL among patients at high risk for cardiotoxicity.

### Goal of the Study

As a first step to closing this gap, we sought to determine considerations for the design and implementation of an mHealth symptom-tracking tool for early recognition of cardiotoxicity in patients with cancer. We explored this issue using the socio-technical systems (STS) framework to model complex interactions between goals, people, processes, infrastructure, culture, and technology.

### STS Framework

Health care systems operate within complex adaptive environments that are constantly evolving, particularly in the high-pressure context of care delivery [27]. This dynamic setting makes the implementation of Health Information Technology interventions, such as mHealth apps, a formidable challenge. While several conceptual models exist that examine the implementation of technology innovations in health care, they are usually limited in scope [27]. Importantly, many of these models fail to address the intricate relationships that exist between different dimensions of implementing technology innovations in a health care setting such as those related to the deployment of an mHealth app.

**Figure 1:**
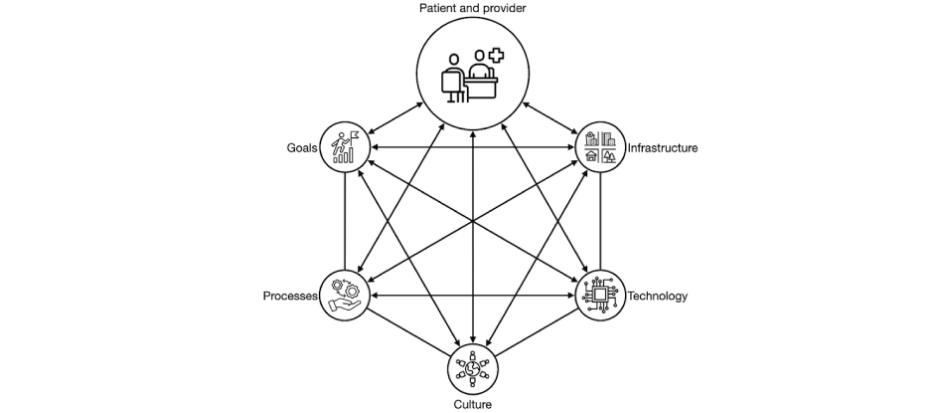
STS framework. STS: socio-technical systems.

The STS framework offers a systems-oriented perspective on organizations (Figure 1). Within this framework, readiness and implementation are considered within the context of various interconnected subsystems. Overcoming barriers to the implementation of digital tools in health care, including mHealth apps, involves addressing challenges like organizational readiness and the alignment between the tool and existing workflows [28]. Sociotechnical theory asserts that the successful implementation of mHealth interventions is contingent on both technical factors, such as ease of use, and social and organizational factors, including leadership support.

Sociotechnical theory posits that the successful implementation of mHealth is a function of both technical (eg, ease of use) and social and organizational factors (eg, leadership support) [29-31]. The associated STS framework [32] is composed of six domains [33]: (1) goals: This encompasses performance metrics and objectives that guide the implementation efforts. (2) People: This refers to individuals within the system, including their attitudes, behaviors, skills, and competencies. (3) Infrastructure: Physical and financial assets necessary for the implementation, ensuring adequate resources. (4) Technology: The technological components, tools, and equipment required for the intervention. (5) Culture: Shared norms, beliefs, and values that influence the organizational environment. (6) Processes: Work practices and organizational structure that influence how the intervention is integrated.

We selected this framework to explore the design and possibility of implementation of the app into a health system. Further, our approach emphasizes a strong focus on user-centeredness. Specifically, we have applied the STS framework in relation to patients, who are the primary end users. Their perspectives offer valuable insights into critical factors such as design preferences, expected features, and the willingness to adopt the app. Additionally, we have also engaged health care providers, who play a significant role not only in receiving the app’s data but also in the early identification of cardiotoxicity. By considering viewpoints from both patients and providers, our study aims to provide a deeper understanding of how mHealth implementations can be aligned with the specific needs of these essential stakeholders.

Our research addresses a significant gap in the current literature. While numerous studies have separately investigated the viewpoints of patients or providers in the context of mHealth, the synergistic interaction between these perspectives has often been overlooked [34-36].

Our study seeks to bridge this gap by acknowledging the essential interdependence between the perspectives of patients and providers within the intricate domain of mHealth. By using this approach, we intend to enhance the comprehension of effectively integrating the sociotechnical complexities of mHealth with the distinct requirements of these crucial stakeholders.

## Methods

### Participants

#### Providers

Leveraging a large, university-affiliated comprehensive cancer center, we recruited cardiotoxicity providers from our health system. In 2022, the health system managed over 58,000 patient admissions and over 2.25 million outpatient visits. Using convenience sampling, we sought clinical providers who worked with patients at risk of cancer treatment-related cardiotoxicity (ie, board-certified cardiologists, oncologists, radiation oncologists and cardio-oncologists).

#### Patients

We recruited patients using ResearchMatch.org and via convenience sampling. Research Match (Vanderbilt University) is a web-based service that connects researchers from over 200 US academic institutions to volunteers, living in the United States, and who are willing to participate in research studies. Volunteers sign up and create a profile by providing their demographics, contact information, and information about their health. Researchers can search the Research Match database for registered volunteers who match the study inclusion criteria. For this study, we required that patients were older than 18 years of age with the capacity to give consent, were English-speaking, owned or used a smartphone, and had a cancer diagnosis. To improve generalizability, we did not require participants to be part of our institution or reside in a specific part of the United States. Participants who fit our criteria and indicated that they were interested in participating were contacted by a study team member via telephone to confirm all inclusion criteria and eligibility, and to set up a time for a web-based meeting to conduct the study procedures.

### Ethical Considerations

This study was approved by The Ohio State Cancer Institutional Review Board (#2021C0018). All participants consented verbally before any study procedures. Patients were also provided the consent document via email before beginning any study procedures. All study documents were deidentified and files were saved to a secure server behind institutional firewalls. Study documents were saved with the date and time of the interview rather than participants’ names, and names were replaced with codes (eg, Participant 1 and cardiologist 1).

### Procedure

#### Providers

We conducted 15- to 30-minute semistructured web-based interviews with providers. Topics were related to the STS domains and included: the provider’s role (eg, “What type of cancer patients do you work with?”), opinions on app design (eg, features; anticipated challenges with patient uptake; eg, “If patients were to use an app to help you understand and manage their condition, what features do you think would be most helpful?”), signs and symptoms indicating cardiotoxicities (eg, “What pieces of information (eg, symptoms) would you like to see?”), and electronic health record (EHR) integration (eg, “If the app could push data to the patients’ EHR, would this be helpful to you?”). Demographic information, including role, how long participants had been in their role, and the type of cancer the participants treated, was asked during the interview. The interview guide was codeveloped by a team member experienced in qualitative methods and a cardio-oncology physician. The cardio-oncology physician team member piloted the guide before other participants were interviewed.

#### Patients

We conducted 30-minute semistructured web-based interviews. The interview guide was developed by the research team and focused on 3 primary topics that were related to STS domains: the patient’s cancer, treatment, and symptoms (eg, “What information about your condition do you keep track of and report to your doctor?”); perspectives on the potential app (eg, “How could this app work best for you? For example, would you like to receive reminders or notifications from it?”); and positive and negative prior experiences with mobile apps (eg, “I want you to think of an app you have used or are currently using that you have particularly enjoyed. Can you describe features of the app that you particularly like [liked], that are [were] easy to use, or that help [helped] achieve what you want [wanted]?”). During the interview, the following demographic information was collected: age, gender, race, ethnicity, cancer treatment status (active treatment or survivor), cancer type, and when cancer was diagnosed. Interviews were recorded and transcribed. Patients received a digital gift card (US $25) for participation in this phase.

**Figure 2:**
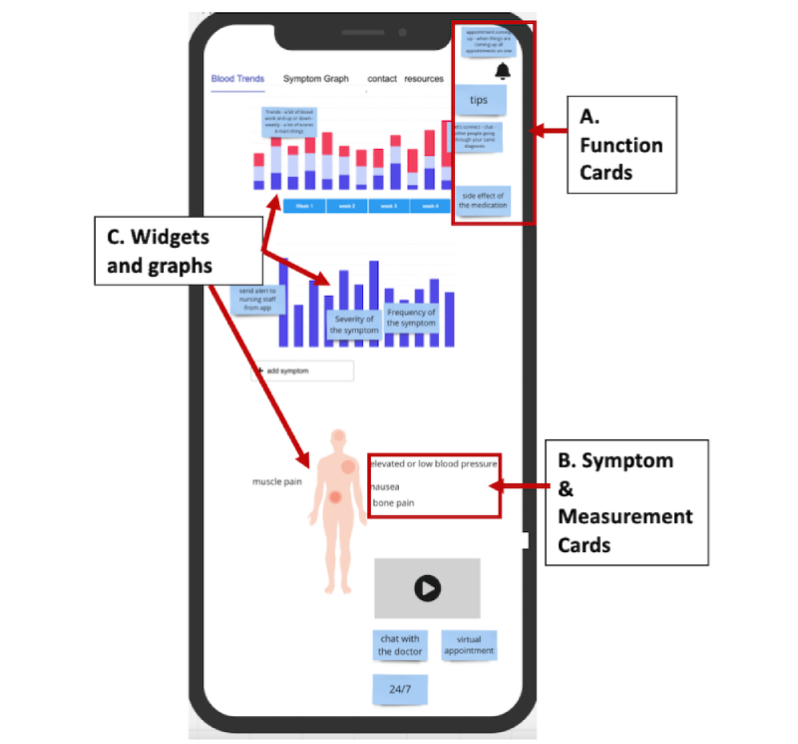
Example of patient app design from co-design process.

We also subsequently engaged patients in a 60-minute co-design process using a web-based whiteboard, wherein they interfaced with a blank mobile app screen to design their ideal app display and mock-up a prototype app. Each co-design session involved only 1 participant. These sessions were facilitated by a team member experienced in co-design. Working virtually with the facilitator, participants were provided a link to the web-based whiteboard and were asked to share their screen. There, they were given several precompleted function cards (sticky notes describing features such as “send alert to message staff from app,” “tips,” “chat with the doctor,” etc); symptom and measurement cards (textboxes and potential icon displays of the symptoms to be tracked in the app); and widgets and graphs (allowing for choices between bar charts, line graphs, choropleth diagrams, pie charts, and several other graph types). They were informed that they could drag any of these options into the blank screen and design their ideal app for symptom tracking, including their desired graphical display of symptoms, and they were encouraged to add in any other app features that they would like to see (see Figure 2). They could also add their own blank sticky note to indicate a different feature, look, or option that was not provided. Patients received a digital gift card (US $50) for participation in this phase. The co-design sessions were recorded and transcribed.

### Analysis

#### Provider Data

Provider data were thematically analyzed [37,38] following a combined inductive-deductive process [39]. First, we developed a codebook with code categories predefined, based on sociotechnical theory to consider both technical (eg, app features) and social and organizational factors (eg, workflow). The codebook subsequently evolved wherein new codes emerged and definitions were clarified [40,41]. The coding team was trained and led by the lead author, who is experienced in qualitative methods and analysis. Coding was done independently, with each transcript coded by 1 team member, but the principal investigator reviewed all coding.

#### Patient Data

Patient data were analyzed following an inductive approach using thematic analysis [37,38]. We reviewed the interview and co-design transcripts and coded comments into common themes (including app features desired, such as medication tracking, ability to contact provider, and other themes, such as the desire for the ability to customize the app). These data were supported with the patient app mock-ups and were used together to determine frequencies of desired features and options for the app, the desired look of the app, patient demographics, and patient symptoms. Noteworthy quotations that helped contextualize these findings were marked for potential inclusion in the study. Each transcript was coded by 1 team member, with the principal investigator reviewing all coding.

## Results

### Demographics

#### Providers

We interviewed 11 providers, including 3 cardio-oncology physicians, 2 oncologists, 3 oncologists or hematologists, 1 radiation oncologist, and 2 general cardiologists. Providers included cardiotoxicity fellows (n=2) and attendings (n=9).

#### Patients

A total of 6 patients participated, with 5 (83%) participating in a semistructured interview, and 6 (100%) in co-design; including 67% (n=4) with multiple active cardiotoxicity symptoms. Demographics are shown in Table 1.

**Table 1:** Patient demographics.

Variable		Values
Age (years), mean (SD; range)		55 (9.68; 46-66; 2^a^)
**Race or ethnicity, n (%)**		
	White	4 (67)
	Black	1 (17)
	Not reported	1 (17)
**Gender, n (%)**		
	Male	1 (17)
	Female	5 (83)
**Cancer type, n (%)**		
	Breast	5 (83)
	Prostate	1 (17)
**Treatment status, n (%)**		
	Active treatment	1 (17)
	Survivor	5 (83)
**Treatment type^b^, n (%)**		
	Chemotherapy	4 (67)
	Radiation	2 (33)
	Surgery	5 (83)
	Other	1 (17)
**Potential cardiotoxic treatment-related symptoms experienced^b^, n (%)**		
	Chest pain or tightness	0 (0)
	Shortness of breath	1 (17)
	Heart palpitations	2 (33)
	Abnormal heart rate	1 (17)
	Abnormal blood pressure	1 (17)
	Edema	2 (33)
	Lightheadedness	1 (17)
	Syncope	0 (0)
	Excessive fatigue	2 (33)
	Total number of patients reporting >1 symptom	4 (67)
**Mobile health used^b^, n (%)**		
	Patient portal	4 (67)
	Health apps	3 (50)
	Other	1 (17)

^a^Number of participants who did not report.

^b^May sum to >6 as some participants reported multiple.

### Providers’ Perspectives: Current State

Table 2 describes the current state of cardiotoxicity symptom tracking and reporting at the institution, mapped onto the STS framework. There is no systematic process for patients to report potential cardiotoxicity symptoms. Rather, it is up to patients to recognize and choose to contact their provider via phone or patient portal. Alternatively, symptoms may go unreported until a patient has a clinic visit, causing delays in cardiotoxicity recognition. Providers also reported that patients may not recall symptoms during their appointment, and thus it may go unreported entirely. There is no standardized process for incorporating symptom information into the EHR, and no process for providers or staff to regularly manage symptom reports. Cardiologist 1 expounded upon this by describing a potential barrier to such a process, wherein providers and staff would need billable time for this:

...nurse practitioners could [receive symptom reports]. They're fully capable of it, but it's if it's all non-billable time...I mean with the way that the health care system is designed right now...pretty much if you're not billing...

**Table 2:** Current states of potential serious cardiotoxic symptom capture and treatment.

Domain	Current state: patients report symptoms at visit or call in	Representative quotations
Goals	Cardio-oncology issues reported at appointments	*If somebody comes to my clinic every three months...symptoms...might not have risen to the test threshold of them calling you...[and] that day they might not be feeling shortness of breath [and therefore it goes unreported]*. [Cardio-oncologist 1]
Culture	Organization’s EHR^a^ largely precludes integration of app data into EHR	*...if you could get Epic to play along, sure, but...there's going to be some barriers...* [Oncologist 1]
People	Some patients are higher risk for cardiotoxicity and would benefit from providers having more timely information	*Groups of patients that have received large doses of anthracyclines, combination of cardiotoxic chemotherapy plus radiation, high dose radiation...who we know are at an increased risk and we want to kind of keep a closer eye on...having more information may be helpful to their care.* [Cardiologist 1]
Technology	Current technology is not amenable to early reporting of symptoms	*Symptoms outside of those acute encounters is going to be beneficial for patient care.* [Cardiologist 2]
Infrastructure	Current infrastructure requires patients to actively choose to contact providers about symptoms outside of appointments	*Our patients use MyChart on Epic, and if there's something serious they just put in a message and so someone from our team receives it.* [Oncologist/hematologist 3]
Processes	No current process for a provider or staff member to receive regular reports of symptom data	*From a patient perspective, it would be great to know that someone was like watching your vital signs all the time, but from a physician standpoint we just don't have the resources to do that.* [Oncologist 1]

^a^EHR: electronic health record.

### Providers’ Perspectives: Goal State

Table 3 describes the goal state of a more robust, timely cardiotoxicity symptom reporting and recognition process leveraging mHealth. Providers posited it would give patients an alternative method to report symptoms, and patients would be motivated to use the app if they understood that it was a faster way to communicate with their provider. Providers suggested that the app could also prompt discussion during appointments:

[with the app] if I would have seen their click, the shortness of breath button 200 times in between the previous visit and now and ask them...like, ‘you seem to be reporting this quite a bit...Is it something which you're really feeling or you not just feeling it today?’ That might be a question which I might then ask, which I would have not asked before.

Cardio-oncologist 1

Providers cautioned that to ensure adoption, the app should be straightforward and simple, and not ask too much of patients, for example,

That may be a bit discouraging, like if you had them log their blood pressure every hour or something...if there's a lot of busy work that the patients having to put into the app, that may be a barrier.

Cardiologist 1

Providers suggested that patients should be able to customize the app, such as whether they wanted it to send them reminders to report symptoms.

**Table 3:** The goal states to improve the integration and effectiveness of mobile apps into cardio-oncology care.

Domain	Goal state: addition of mobile app to facilitate earlier recognition	Representative quotations
Goals	Earlier recognition of emergent or worsening cardio-oncology issues	If someone's having a side effect of the treatment you want to know about it as soon as possible to help prevent further harm. [Oncologist 1] When it would be helpful for me to get that information? I think probably realistically in real time, you know as soon as we encounter a major problem. [Oncologist/hematologist 1] If they had some event that happened between appointments and they just were like ‘oh, I wouldn't get through to somebody. So I'd rather just log in through the app,’ that would be helpful. [Oncologist/hematologist 3]
Culture	Working within cultural constraints to incorporate patient-facing technology into EHR^a^ without writing to EHR	As long as it doesn't interfere with something...algorithms can mess up with each other, so as long as it doesn't disrupt the functioning of our EHR, I think it should be helpful. [Oncologist/hematologist 2]
People	Getting patient buy-in to use the app will be crucial for this to work	That's going to kind of be a game changer for patients if they understand that… this is potentially a faster and more efficient way for me to communicate with my doctor or their office. It changes the calculus as far as how much effort somebody’s gonna put into things. [Cardio-oncologist 2]
Technology	Mobile app to track regularly reported symptoms Keeping the app simple is important Patients should have options to tailor the technology (eg, turn off reminders)	People who aren't...technically savvy are still willing to use [technology]...Just you can't make it overly complicated because I think people get overwhelmed fairly quickly. [Radiation oncologist 1] Maybe like tailoring...like they can toggle the reminders on or off if there were anxious person that doesn’t want a reminder...turn it off. [Oncologist/hematologist 3]
Infrastructure	Work within existing infrastructure to facilitate transfer of data from mobile app to providers or staff (eg, EHR inbox)	...if it could be linked to MyChart. It would beep or send an alert to the MyChart that at this time, patient had went into >30 seconds a-fib or something like that... [Oncologist/hematologist 2] It’d be nice if it would go to my in-basket, and I would get paged at the same time. Just so someone looks at it quickly if it's a serious event. [Radiation oncologist 1]
Processes	App facilitates more timely and efficient symptom reporting Looping in all providers (cardiology, oncology, and primary care) is crucial	I think eventually clinically this is going to be potentially used in the same way we use MyChart. Right now for Epic, basically patients are told at the time of their initial engagement with the office, even before they speak to the physician, that they have this electronic mode to communicate with more efficiently and they don't have to make phone calls every time they have an issue. [Cardio-oncologist 2] ...maybe like a co-management model with oncology and cardio-oncology...Even if cardio-oncology is getting that data, I still have to decide whether to hold their treatment or not...I can't think of a situation where it would work solely with cardiology leading it. [Oncologist 2] I think we would, between the cardiologist and the oncologist, figure out what needed to be adjusted together...can we hold this oncology medication? Is it safe to or not? So those are conversations we would have. [Oncologist/hematologist 3]

^a^EHR: electronic health record.

Providers mentioned that oncology, cardiology, and primary care should all be involved when a patient shows potential signs of cardiotoxicity, to allow them to codevelop a plan. This is explained here:

...it's really important that you guys think about oncology being in the mix for sure. Also, primary care...or if they have a previous relationship with the cardiologist...thinking of a team-based model rather than their results going to one person.

Oncologist 2

In terms of EHR integration, the institution requires that data from external apps be reviewed by a clinician before adding it to the patient record, challenging the idea of a full EHR-app integration. Providers described how a similar goal could be achieved while working within this constraint, such as having concerning patient symptom data trigger an alert in the patient portal or provider in-basket.

### Providers’ Perspectives: Signs and Symptoms to Report in the App

Providers indicated that signs and symptoms indicative of cardiotoxicity, that should be added to the symptom-tracking app, included: chest pain or tightness, shortness of breath, heart racing or palpitations, syncope, lightheadedness, edema, and excessive fatigue.

### Patients’ Perspectives: Current State

There was no current standardized process for patients to report symptoms. Most patients indicated waiting until appointments to discuss symptoms, unless they felt that it was urgent, in which case they would call or message their provider’s office. For example, 1 patient stated (Table S1 in Multimedia Appendix 1):

I don't write [my symptoms] down. I just know, OK, it started a couple weeks ago...and I kind of just keep a mental note, and then if I feel like it's something I need to tell [the doctor], then I do

### Patients’ Perspectives: Goal State

As shown in Table 4, all patients indicated an interest in tracking symptoms via a mobile app. Participants foresaw using these data in 2 ways: first, by allowing them to see trends in their symptoms and symptom severity, and second, to communicate symptoms to their provider. Toward the former, patients desired a bar chart display to see their (cardiotoxicity) symptoms over weekly, monthly, and yearly time periods. Toward the latter, participants expressed interest in sending their symptoms and related questions via the app, to get their providers’ feedback and interpretations. Some also hoped that the app could alert them and their provider if a concern needed to be addressed immediately.

**Table 4:** Patient preferences for the features of the app.

Patient preferences	Values, n (%)
Desire to track symptoms	6 (100)
See trends in symptoms: weekly, monthly, and yearly	6 (100)
Track symptom severity	6 (100)
Use app to communicate with or contact provider	6 (100)
Additional support features: community of other patients or survivors, educational resources	6 (100)

Patients indicated that an app would need to be easy to use. For example, when asked about prior apps that were disliked, 1 patient indicated the following:

Usually because I felt they were too complex. It was too much work to use them. For instance, to get to a certain feature that you wanna use, maybe I need to go five steps instead of two steps. It was just too cumbersome, or it takes too much time...time is important to me, I just delete those kind of apps.

On the flip side, patients indicated liking apps that were simple. For example, when asked about an app that the patient liked, they indicated:

I think because it was simple, and it just was inviting. Like the colors are inviting and also has prompts for you...it was easy.

### Patients’ Perspectives: Additional Features Suggested in App

Participants indicated an interest in additional app features, including educational resources and the ability to build a community of other patients with cancer and survivors (eg, discussion board), as exemplified by 1 patient:

It would be nice if there was a good safe go-to place where you could find out more information from maybe other cancer survivors...it's nice to hear what doctors really have to say but...if there's like one other person who is experienced in that same symptom and you can have a conversation with them, that's kind of nice.

**Figure 3:**
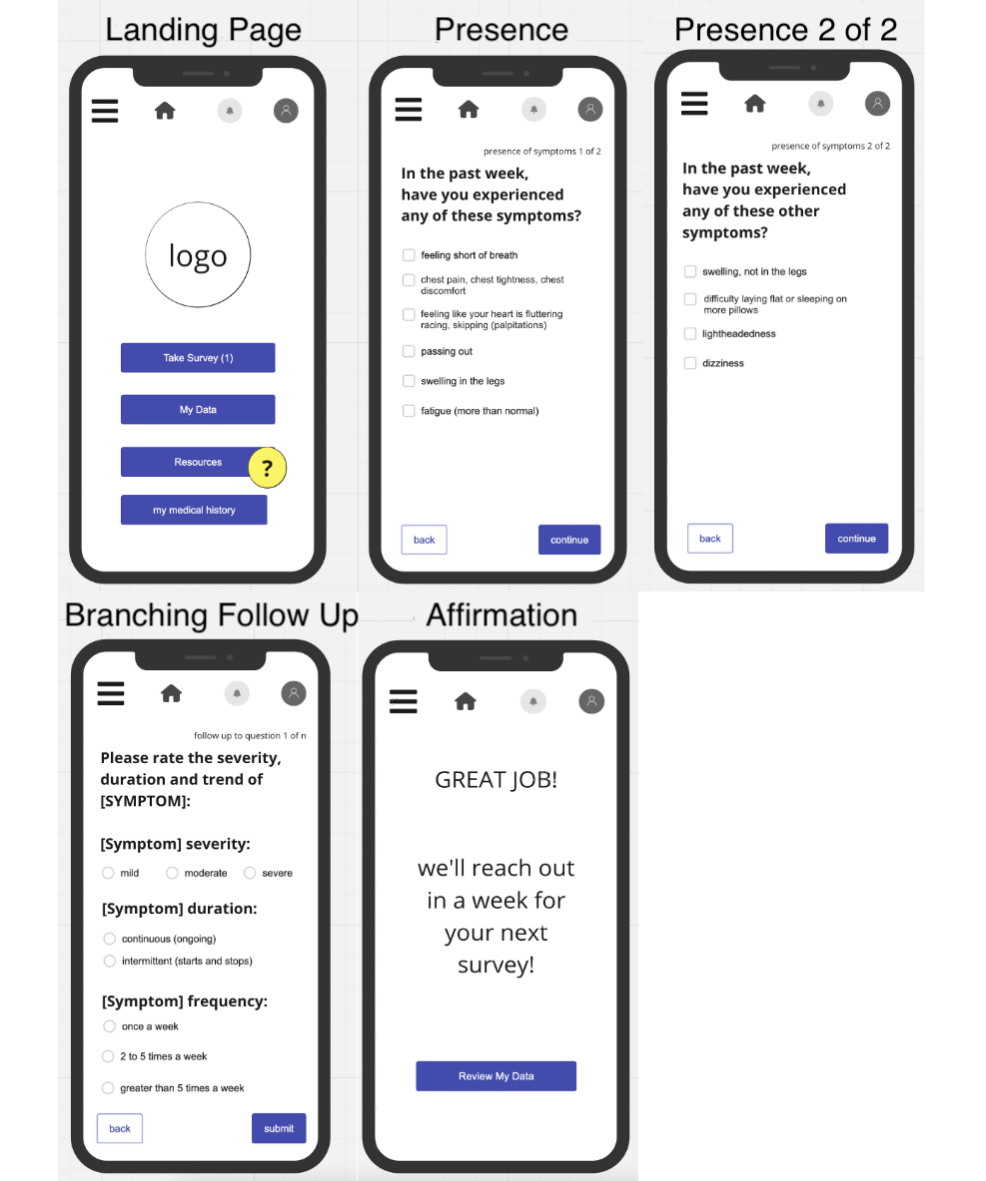
Display of front page of app and symptom logging feature based on patient input and provider symptom list.

**Figure 4:**
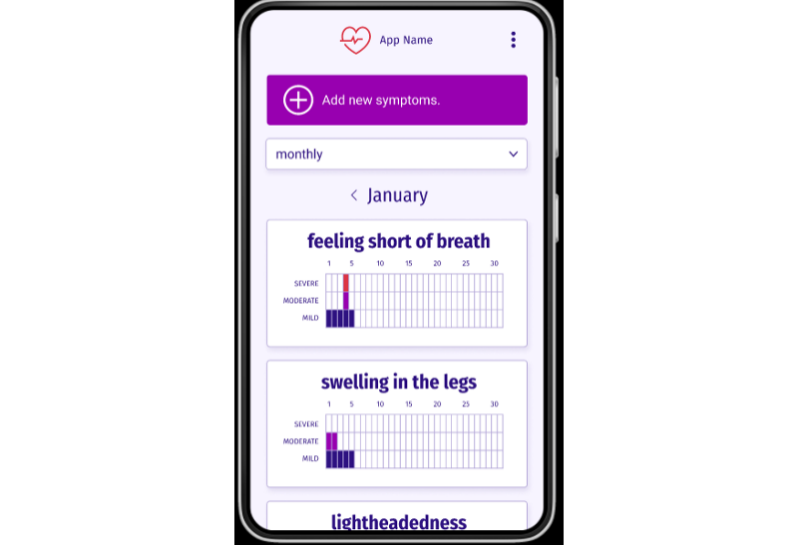
Finalized display of chart feature based on patient input.

Figures 3 and 4 show the app design resulting from the co-design process. Figure 3 displays the front page of the app, as well as the symptom tracking feature wherein participants can indicate and rate their symptoms. These ratings would ideally serve 2 purposes: first, to provide an early alert to providers of concerning symptoms indicative of potential cardiotoxicity via regular transmission of these data to clinical staff and second, to be maintained within the app to inform a chart to allow for visualizing patterns over time. Toward the latter, Figure 4 shows the graph display preferred by patients (a bar chart style) which allows patients to view their symptoms and severity of symptoms over time. Patients indicated being interested in viewing this for their own knowledge, and both patients and providers indicated interest in using these graphs during clinic visits to inform clinical care.

## Discussion

### Principal Findings

This study evaluated providers’ and patients’ perspectives on the design and implementation of a novel cardio-oncology mHealth app (for symptom reporting and early cardiotoxicity recognition). Providers and patients expressed positive attitudes and described barriers to early cardiotoxicity reporting. Findings suggest that the app should be designed for patients with cancer at higher risk for cardiotoxicity, should motivate patients to log symptoms, and should allow providers to collaboratively comanage symptoms efficiently.

A key factor of success identified is to motivate the users to engage in symptom-logging behaviors for an extended period [42]. Both patients and providers suggested that designing the app to be simple, and to allow patients to tailor the app to their preferences, would facilitate app engagement. Further, providers suggested that explaining the benefits of the app (eg, more efficient communication) would help motivate patients to adhere to symptom logging.

Our findings are in agreement with prior work [34,35] showing that digital solutions have the potential to address unmet needs, such as facilitating symptom monitoring, detecting adverse effects, improving cancer self-management, and empowering patients. However, similar to prior work [42], providers suggested that a lack of billable time assigned to monitoring and managing app symptom reports would be a barrier.

Similar to prior work [43], providers expressed that it would be ideal to incorporate app data into the EHR to enhance patient-centered care. However, cultural and infrastructure-related barriers complicate this. Alternative modes (eg, in-basket messages and linking to the patient portal) could be considered. Providers also indicated that the app could facilitate face-to-face doctor-patient communication (eg, reviewing symptom logs during appointments). Data generated and recorded from mHealth apps may be considered billable or admissible to the patient health care record, given the potentially significant influence on patient outcomes. This would leverage the current insurance bundled health care delivery mode, with the patient’s desire for quick and effective ways to communicate with providers via verifiable and self-reported information. Practically, patients may be allowed to opt-in to having their mHealth data recorded within the EHR (eg, similar to Epic’s “MyChart” system).

Providers have previously expressed concerns that during critical situations, patients may report severe symptoms to an app, expecting that it is being actively monitored [35]. This concern seems well-founded based on our data. It is unlikely that this would be the case without additional personnel for this role. Thus, we suggest the app include notifications and that urgent or severe symptom should be reported another way (eg, by calling the provider’s office, going to the emergency room, or calling 911).

The STS framework played a significant role in understanding the implementation of the cardio-oncology mHealth app. The six domains of the STS framework were instrumental in guiding our study: (1) goals: The study focused on understanding performance metrics and objectives, aiming to design an app that effectively addresses the needs of both providers and patients for early cardiotoxicity recognition. (2) People: Providers and patients—the pivotal end users—were the lens through which the STS framework was operationalized in this study. Their attitudes, behaviors, and competencies shaped the app’s design. Providers expressed the need for patient motivation to engage in symptom-logging behavior over an extended period, emphasizing simplicity and personalization. Patient adherence was seen to be fostered by explaining the benefits of efficient communication enabled by the app. (3) Infrastructure: The study recognized the significance of sufficient physical and financial resources for implementing the app effectively. Challenges in allocating billable time for monitoring app reports were acknowledged as a potential barrier, indicating a need for resource allocation. (4) Technology: The technological components required for the mHealth app were central to the study’s evaluation. It was emphasized that the app should not replace urgent traditional communication methods for severe symptoms, suggesting a need for clear technological boundaries. (5) Culture: Shared norms and values influence the organizational environment. Integrating app data into the EHR was identified as a cultural challenge at this institution. As part of a broader organizational culture around information security [44], the institution had organizational policies that disallowed external apps to write data to the EHR. Alternative methods, like in-basket messages or patient portal links, were proposed. (6) Processes: Work practices and organizational structure were taken into account. The study highlighted the potential role of the app in facilitating doctor-patient communication, allowing for symptom review during appointments and potentially integrating app-generated data into patient records.

Consistent with prior research, the study found that mHealth apps have the potential to address unmet health care needs by enhancing symptom monitoring, supporting patient self-management, and improving communication. However, the study also underscored challenges related to resource allocation and integration with existing health care practices. The findings suggest that for successful implementation, the app should be carefully tailored to address these technical, organizational, and behavioral considerations.

Concerns raised by providers about patients expecting active monitoring of severe symptoms through the app were acknowledged. To address this, we recommended incorporating notifications within the app to guide patients on reporting urgent or severe symptoms through appropriate channels, such as calling the provider’s office, seeking emergency care, or dialing 911. The STS framework facilitated a comprehensive understanding of the app’s potential, its challenges, and strategies to ensure successful adoption and use within the health care ecosystem.

### Study Limitations

While this study leveraged a multidisciplinary group of cardio-oncology specialists, the providers were from 1 hospital, and our sample size was small. However, our small sample was largely due to a small population, which was widely represented: participants included 2 out of 3 (67%) of the institution’s cardio-oncologists, and over half of the physicians affiliated with the clinical cardio-oncology program. Implementation in other types of institutions should also be explored, as sociotechnical factors likely vary in relation to organizational size and resources. This evaluation focused on cardiotoxicity given the serious consequences to patients. In addition, most transcripts were coded by only 1 individual, although all coding was reviewed by the first author. Further, patient recruitment was challenging, and thus we were limited in obtaining information from patients who have experience with more novel cancer therapies with higher rates of cardiotoxic effects. In future work, we will be better resourced to selectively recruit this specialized group. Regardless, 67% (n=4) of our patients sampled did indicate having 1 or more indicators of potential cardiotoxicity related to cancer therapy, with 1 reporting having developed a cardiac condition. We also acknowledge that the decision to recruit patients from outside the institution may have limited what we were able to learn about how to implement the app in this particular organization.

This study focused solely on symptom trackers or monitors. However, we acknowledge that some patients see cardiotoxic injury well before the onset of symptoms. We also note that emerging biomarkers, including blood or imaging-based biomarkers for example, may further advance the ability to detect and track cancer treatment-induced cardiotoxicity well before the onset of clinical symptoms and the manifestation of more advanced disease. In future work, we plan to consider the concurrent leveraging of other clinical tests with this app. With many at-home, single-lead electrocardiogram (ECG) machines and wearable smartwatches with single-lead ECG functionality, it may be possible to incorporate such data into an app. These data could be logged with other clinical data in the app alongside home blood pressure readings and heart rate monitoring data from wearable technology to facilitate rapid triage of new, potentially worrisome symptoms of cardiotoxicity. We will consider further integration with emerging smartwatch application data.

### Clinical Implications

mHealth can play a role in early recognition of clinical complications of cancer treatment, such as cardiotoxicity. However, incorporation of mobile apps into clinical care requires working through persons, systems, and technology-related barriers to ensure success.

### Conclusions

Providers and patients perceived that a patient-facing symptom-reporting app would be beneficial to increase early recognition of cancer treatment-related cardiotoxicity, as current processes are perceived to lead to delays in recognition and treatment. However, sociotechnical barriers include the lack of a process for multidisciplinary providers to have allocated time to review app data and collaborate on care plans, infrastructure-related challenges limiting how mHealth data can be incorporated into the EHR, and designing an app that is simple and tailored to patients to motivate use.

## Appendix

Multimedia Appendix 1 Patient perceived barriers and needs for cardiotoxicity symptom capture.

## Abbreviations

ECG: electrocardiogram
EHR: electronic health record
mHealth: mobile health
QOL: quality of life
STS: socio-technical systems
