# Supplementary material for: Exploring the Incorporation of a Novel Cardiotoxicity Mobile Health App Into Care of Patients With Cancer: Qualitative Study of Patient and Provider Perspectives
Source: JMIR Cancer. 2023 Dec 12;9:e46481. doi: 10.2196/46481 (PMC10751627; doi:10.2196/46481)
Supplement: Multimedia Appendix 1 [file cancer_v9i1e46481_app1.docx]

| **Current State: Patients reported challenges** | **Representative Quotations** |
| --- | --- |
| Challenges in reporting important cardiotoxicity symptoms at appointments | “I don't write [my symptoms] down. I just know, OK, it started a couple weeks ago … and I kind of just keep a mental note, and then if I feel like it's something I need to tell [the doctor], then I do”- Patient 1 |
| Reasons for not using apps for cancer (cardiotoxicity) care | “Usually because I felt they were too complex. It was too much work to use them. For instance, to get to a certain feature that you wanna use, maybe I need to go five steps instead of two steps. It was just too cumbersome, or it takes too much time... So because time is important to me, I just delete those kind of apps”. -Patient 2 |
| Needs and desires from an app to facilitate improved care and outcomes | “It would be nice if there was a good safe go-to place where you could find out more information from maybe other cancer survivors…I mean 'cause it's nice to hear what doctors really have to say but… if there's like one other person who is experienced in that same symptom and you can have a conversation with them, that's kind of nice”. -Patient 3 |
